# Supplementary material for: TP53 Mutation Predicts Worse Survival and Earlier Local Progression in Patients with Hepatocellular Carcinoma Treated with Transarterial Embolization
Source: Curr Oncol. 2025 Jan 18;32(1):51. doi: 10.3390/curroncol32010051 (PMC11764326; doi:10.3390/curroncol32010051)
Supplement: Supplementary file 1 [file curroncol-32-00051-s001.zip › curroncol-3321851-supplementary.pdf]

Current Oncology

MDPI

Table S1. Prognostic factors of improved overall survival, multivariate analysis.

	Multivariate Cox Analysis		
	**HR**	**95% CI**	**p-value**
TP53 status (Wild-Type vs Mutant)	0.73	0.41-1.32	0.300
AFP (ng/mL) (≤200 vs >200)	0.43	0.23-0.78	0.006
ECOG PS (0 vs 1 or 2)	0.51	0.23-1.12	0.093
BCLC Stage (A or B vs C)	0.99	0.46-2.12	0.983
Tumor Number (1 vs >1)	0.56	0.26-1.19	0.130

 https://doi.org/10.3390/curroncol32010051
